# Measuring Progress Toward Universal Health Coverage: Does the Monitoring Framework of Bangladesh Need Further Improvement?

**DOI:** 10.7759/cureus.2041

**Published:** 2018-01-08

**Authors:** Rajat Das Gupta, ASM Shahabuddin

**Affiliations:** 1 James P Grant School of Public Health, BRAC University

**Keywords:** universal health coverage, monitoring framework, bangladesh

## Abstract

This review aimed to compare Bangladesh’s Universal Health Coverage (UHC) monitoring framework with the global-level recommendations and to find out the existing gaps of Bangladesh’s UHC monitoring framework compared to the global recommendations. In order to reach the aims of the review, we systematically searched two electronic databases - PubMed and Google Scholar - by using appropriate keywords to select articles that describe issues related to UHC and the monitoring framework of UHC applied globally and particularly in Bangladesh. Four relevant documents were found and synthesized. The review found that Bangladesh incorporated all of the recommendations suggested by the global monitoring framework regarding mentoring the financial risk protection and equity perspective. However, a significant gap in the monitoring framework related to service coverage was observed. Although Bangladesh has a significant burden of mental illnesses, cataract, and neglected tropical diseases, indicators related to these issues were absent in Bangladesh’s UHC framework. Moreover, palliative-care-related indicators were completely missing in the framework. The results of this review suggest that Bangladesh should incorporate these indicators in their UHC monitoring framework in order to track the progress of the country toward UHC more efficiently and in a robust way.

## Introduction and background

Universal Health Coverage (UHC) is a revolutionary concept adopted by World Health Organization (WHO) and recently incorporated as a target in Sustainable Development Goal (SDG) [1-2]. The basic concept of UHC is, without suffering any financial hardship, all people should have access to quality health care according to their needs. UHC has three dimensions: population coverage, quality service coverage (including preventive, curative, promotive, palliative, and rehabilitative health care services), and financial risk protection [3-5].

UHC is a crying need in the current age, where half of the world population is deprived of the service they need and only half of the covered services has financial protection [6]. The situation is the worst in all dimensions of South East Asia, including Bangladesh [3-7].

In Bangladesh, an inequity exists among the health indicators of different socio-economic groups and geographical locations [8-11]. Currently, in Bangladesh, among the total health expenditure, 64% is paid out of pocket [7]. Every year, almost 4% of the population is impoverished due to high out-of-pocket expenditure [12-13]. Catastrophic health expenditure is highest in Bangladesh among the countries of the Asia Pacific region [14]. Therefore, introducing and implementing the concept of UHC is necessary for Bangladesh. The Government of Bangladesh (GOB) is determined to achieve UHC, which is also manifested by the recent adoption of the UHC progress monitoring framework [2].

Measuring progress toward UHC is necessary to know whether the country is on the right track to achieve UHC. Measuring and monitoring the progress will enable policymakers to identify the gaps and help them plan future activities [3]. Therefore, it is very important to create an effective framework to measure and monitor the progress of UHC.

Considering the importance of the issue, this review aimed to explore the UHC monitoring approaches undertaken and the recommendations at the global level, compare those approaches, recommendations, and challenges with the UHC progress monitoring framework adapted by Bangladesh, find out if any gap exists in Bangladesh’s UHC progress monitoring framework, and address those gaps for further improvement.

## Review

Methods

A thorough search of the relevant literature published and available in PubMed and Google Scholar was conducted using the preferred reporting items for systematic reviews and meta-analyses (PRISMA) checklist independently by two researchers (RDG and ASMSU) [15]. The following search terms were used: ‘Universal Health Coverage,’ ‘UHC’ in combination with ‘global monitoring,’ ‘global monitoring framework,’ ‘measurement,’ ‘Bangladesh,’ and ‘country level monitoring.’ In addition, we manually searched gray literature from the Ministry of Health and Family Welfare (MOHFW), Directorate General of Health Services (DGHS), and Directorate General of Family Planning (DGFP) of Bangladesh. Those articles in the English language and published between January 1, 2005 (the year the World Health Assembly passed a resolution for addressing universal health coverage) to June 15, 2016, were searched [16].

Relevant information from the selected articles was extracted and put in a matrix. A data analysis was done according to the three dimensions of UHC: service coverage, financial protection coverage, and population coverage or equity measurements [3]. Under each dimension, the global and Bangladesh indicators were listed and analyzed thematically.

Results

Search Results

**Figure 1:**
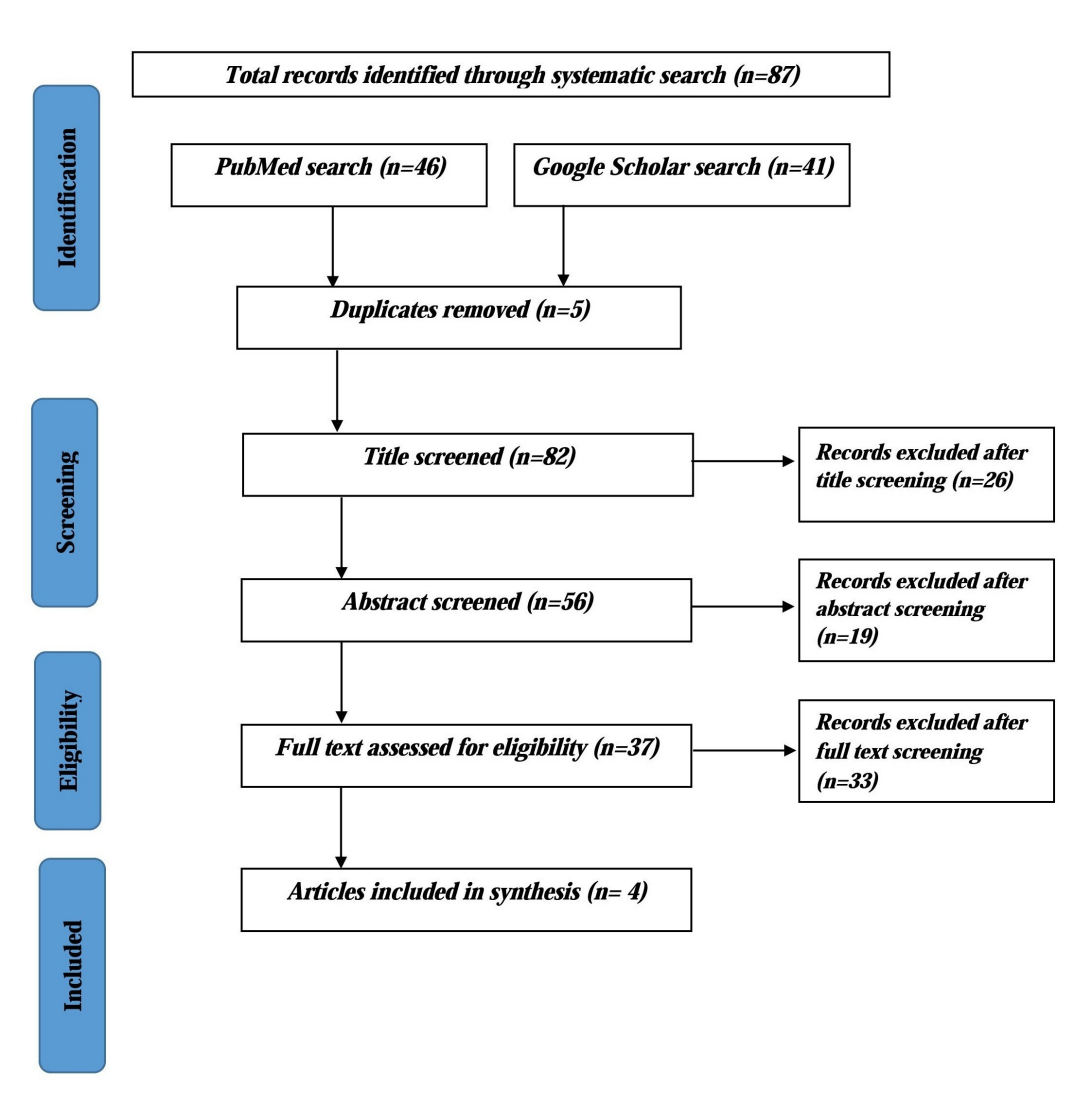
Flow Chart Showing the Literature Search and Selection Process

After a comprehensive search using the selected keywords, we found 87 reports and articles. Among them, five were excluded after duplication checking and 26 were excluded after reading the titles. Another 19 were excluded after reading the abstract and executive summary. A total of 37 articles and reports were retrieved for further, detailed evaluation. Among them, four articles were selected for the final synthesis. Among these four articles, three were reports and one was a journal article (Figure 1). One report was from Bangladesh (Framework for monitoring progress towards universal health coverage in Bangladesh) [2]. The other three reports and articles described the global-level framework [3,5,17].

Principles of Measurement and Targets

Principles of measurement: The Bangladesh framework included 47 indicators, including inputs (health workforce, infrastructure, medicines, health information, and research and health care financing), outputs (service access and readiness and service quality and safety), outcome (coverage of intervention, risk factors and behaviors, and health care financing), and impact (improved health status and health/financial security) [2]. Like the global framework, the Bangladesh framework would track progress using outcome indicators and by measuring the equity perspective [2,5]. 

Targets: Although the global-level framework wanted to achieve the targets of UHC by 2030, Bangladesh set the deadline by 2032 [2,5]. The Bangladesh framework aspired to provide everyone, irrespective of socio-economic condition, place of residence, and gender, at least 80% of the essential health services coverage and 100% protection from financial risks while seeking health services [2].

Measurement of Progress Towards UHC

(a) Curative intervention coverage: For curative intervention coverage, Bangladesh incorporated most of the indicators related with infectious diseases (tuberculosis treatment coverage) and non-communicable diseases (diabetic and hypertension treatment coverage) [2-3]. Additionally, indicators related to acute respiratory infection (ARI) treatment coverage were also being added to the Bangladesh framework [2]. Although the global framework suggested antiretroviral therapy coverage, this was not added to the Bangladesh framework [2-3]. Indicators related to mental health and eye health were not incorporated in the Bangladesh framework (Table 1) [2-3].

**Table 1:** Comparison Between the Global and Bangladesh Frameworks UHC: Universal Health Coverage DPT: Diphtheria, Tetanus, and Pertussis Vaccine Petavalent: Diphtheria, Tetanus, Pertussis, Haemophilus Influenza Type B, and Hepatitis B Vaccine

Comparison of the Global and Bangladesh Frameworks	
Global-Level Recommendations	Bangladesh UHC Monitoring Framework
Curative intervention coverage:	
Infectious Diseases: 1. Antiretroviral therapy coverage [3] 2. Tuberculosis treatment coverage [3]	Infectious Diseases: 1. Tuberculosis treatment coverage: Tuberculosis treatment success rate [2]. 2. Acute respiratory infection (ARI) treatment coverage: Case fatality rate among hospitalized ARI cases [2]
Non-Communicable Diseases (NCDs): 1. Hypertension coverage [3] 2. Diabetes coverage [3] Mental health: Depression treatment coverage [3]	Non-Communicable Diseases (NCDs): 1. Percentage of diabetic and hypertensives receiving treatment (Listed under impact indicators) [2] 2. Mental health: No indicator found
Eye Health: Cataract surgical coverage [3]	Eye Health: No indicator found
Preventive Interventions Coverage:	
Reproductive and Newborn Health: 1.Family planning coverage with modern methods [3] 2. Antenatal care coverage [3] 3. Skill birth attendance [3]	Reproductive and Newborn Health: 1. Family planning coverage: Contraceptive prevalence rate [2] 2. Antenatal care coverage: Percentage of pregnant women attending four antenatal care checkups [2] 3. Skill birth attendance: Percentages of institutional delivery [2]
Child Immunization: 1. DPT-3 immunization coverage among one-year-old children [3]	Child Immunization: 1. Petavalent-3 immunization coverage among under one-year-old children [2]
Infectious Diseases: 1. Malaria protection coverage [3]	Infectious Diseases: 1. Malaria protection coverage: Insecticide-treated bed net coverage among malaria endemic zone [2]
Tobacco Use: 1.Prevalence of no tobacco smoking in the past 30 days among adults age ≥ 15 years [3]	Tobacco Use: No indicator found
Non-Health Sector Determinants of Health: 1. Percentage of population using improved drinking water sources [3] 2. Percentage of population using improved sanitation facilities [3]	Non-Health Sector Determinants of Health: 1. Percentage of households with access to safe water [2] 2. Percentage of households with access to improved sanitation [2]
Neglected Tropical Diseases (NTDs): 1. Preventive chemotherapy coverage against neglected tropical diseases (NTDs) [3]	Neglected Tropical Diseases (NTDs): No indicator found
Palliative Care Coverage:	
1. Morphine-equivalent consumption of strong opioid analgesics (excluding methadone) per death from cancer [3]	No indicator found.
Financial Risk Protection:	
1. Catastrophic health expenditures [3] 2. Impoverishing health expenditures [3].	1. Catastrophic health expenditures: Incidence of catastrophic health expenditures (households %) [2] 2. Impoverishing health expenditures: Incidence of impoverishment due to out-of-pocket health expenditures (populations %) [2]
Equity Measurement:	
Following aspects of disaggregation where applicable [3,17]. 1. Economic status 2. Urban/rural residence 3. Gender 4. Education	Three main aspects of disaggregation: 1. Economic Status [2] 2. Urban/ rural residence [2] 3. Gender: Also focuses on the disadvantaged group of the population [2]

(b) Preventive intervention coverage: Bangladesh incorporated most of the indicators recommended by the global framework, including reproductive and neonatal health, child immunization, infectious diseases, and non-health sector determinants of health [2-3]. No indicator was found regarding preventive interventions related to tobacco use and neglected tropical diseases (NTDs) (Table 1) [2-3].

(c) Palliative care coverage: Although monitoring palliative care coverage was recommended by the global framework, Bangladesh did not include that in the UHC monitoring framework (Table 1) [2-3].

(d) Financial risk protection: Bangladesh followed the globally recommended indicators (incidence of catastrophic health expenditures and impoverishing health expenditures) for measuring financial risk protection (Table 1) [2-3].

(e) Equity measurement: Bangladesh followed economic status, urban/rural residence, and gender as part of measuring equity, which is the same as the global framework [2-3]. Furthermore, Bangladesh’s framework focuses on the disadvantaged group of the population (people living in the char and hilly areas, and people belonging to minority communities) as a part of monitoring equity (Table 1) [2].

Discussion

This review compared the UHC monitoring framework of Bangladesh with that of the global community. Coupled with media coverage and the effect of adjacent countries, UHC has been taken into consideration at the policy level in Bangladesh [7]. In 2012, Bangladesh introduced its first health financing strategy with the aim to achieve UHC by 2032 [18]. Later, in 2014, the government of Bangladesh formulated a monitoring framework for UHC [2]. 

This review identified some of the gaps in monitoring service coverage. Bangladesh has adopted UHC monitoring indicators according to its epidemiological context. The HIV-related indicator was excluded and the ARI-related indicator was included due to the low and high prevalence of the diseases, respectively [19-21]. Bangladesh is facing an epidemiologic transition like many other countries with an increasing burden of non-communicable diseases (NCDs) [22-24]. Currently, 61% of all deaths in Bangladesh are attributed to NCDs [25]. Although the global level has recommended NCD (especially diabetes and hypertension) related indicators in the intervention coverage, Bangladesh did not include those indicators; rather, it included them under impact indicators. This is because Bangladesh does not have any full-scale program exclusively for NCDs; rather, these indicators would assess the NCD treatment coverage provided by various health care providers [26-27]. Bangladesh did not have any indicator regarding the monitoring of the mental diseases’ treatment coverage although it has a significant burden of mental diseases [26,28]. A systematic review found that about 6.5% to 31% of Bangladeshi adults were suffering from different types of psychiatric disorders. This burden ranged from 13.4% to 22.9% for the children in Bangladesh [28]. The ignorance of indicators related to mental health is a gap in the UHC monitoring framework of Bangladesh, which needs to be addressed.

The global framework has recommended cataract surgical coverage as an indicator [3]. Although cataract is the main cause (79.6%) of avoidable blindness among the adult Bangladeshi population, the coverage of cataract surgical is currently low (32.5%) in Bangladesh; moreover, there is geographical inequity in treatment coverage [29-30]. Although Bangladesh introduced ‘National Eye Care Plan’ in 2005 followed by several revisions, the national UHC monitoring framework still did not include cataract surgical coverage [29].

Although the global-level framework has recommended to include tobacco quit rate as a UHC monitoring indicator, Bangladesh did not incorporate any indicators related to tobacco quit rate [2-3]. Rather, the framework included tobacco prevalence rate as an indicator [2]. This is may be due to the lack of planned interventions against tobacco use in Bangladesh. Currently, Bangladesh has a huge burden of tobacco use. As per the estimate of a nationwide survey conducted in 2010, 54% of the Bangladeshi population above the age of 25 years was using some form of tobacco [30]. The prevalence rate was even higher (64%) in urban slums, as revealed by a recent study [31]. Bangladesh has an act that bans any sort of advertisement of tobacco products. Recently, Bangladesh has introduced plain packaging of tobacco products [27,32]. Previous findings suggest that the laws against tobacco consumption are not being enacted effectively in Bangladesh [33].

Bangladesh has a burden of several neglected tropical diseases (NTDs), including visceral leishmaniasis (kala-azar), lymphatic filariasis, dengue/dengue hemorrhagic fever, rabies, and snake bite [21,34-36]. Still, NTDs were ignored in the UHC monitoring framework of Bangladesh. Bangladesh has been implementing mass drug administration (MDA) as part of preventive chemotherapy coverage against lymphatic filariasis [21]. Rabies prevention and control programs are also ongoing [21]. These indicators could be included in the UHC monitoring framework to track the situation of such programs and diseases.

The indicator related to palliative care was ignored in Bangladesh’s UHC monitoring framework. Palliative care services are very limited in Bangladesh [26]. With the absence of this indicator, one of the important dimensions of service delivery will remain unknown to policymakers and public health managers.

## Conclusions

Bangladesh’s UHC monitoring framework successfully adopted a majority of the indicators recommended by the global-level UHC framework. However, significant gaps still exist in Bangladesh’s monitoring framework, creating an opportunity for improvement. Indicators related to palliative care, mental health, cataract surgery, neglected tropical diseases, and measurement of service need should be included in Bangladesh’s UHC monitoring framework. An explicitly defined framework with a comprehensive set of indicators will help Bangladesh track its progress toward achieving UHC, and compare its status with other countries.
